# Amphiregulin Exerts Proangiogenic Effects in Developing Murine Lungs

**DOI:** 10.3390/antiox13010078

**Published:** 2024-01-08

**Authors:** Shyam Thapa, Nithyapriya Shankar, Amrit Kumar Shrestha, Monish Civunigunta, Amos S. Gaikwad, Binoy Shivanna

**Affiliations:** 1 Division of Neonatology, Department of Pediatrics, Texas Children’s Hospital, Baylor College of Medicine (BCM), Houston, TX 77030, USA; shyam.thapa@bcm.edu (S.T.); amrit.shrestha@bcm.edu (A.K.S.); monish.civunigunta@bcm.edu (M.C.); 2 Ochsner Clinical School, The University of Queensland Faculty of Medicine, 1401 Jefferson Hwy, Jefferson, LA 70121, USA; n.shankar@uqconnect.edu.au; 3 Division of Hematology and Oncology, Department of Pediatrics, Texas Children’s Hospital, Baylor College of Medicine (BCM), Houston, TX 77030, USA; axgaikwa@texaschildrens.org

**Keywords:** angiogenesis, amphiregulin, fetal murine lung endothelial cells, hyperoxia, bronchopulmonary dysplasia

## Abstract

Interrupted lung angiogenesis is a hallmark of bronchopulmonary dysplasia (BPD); however, druggable targets that can rescue this phenotype remain elusive. Thus, our investigation focused on amphiregulin (Areg), a growth factor that mediates cellular proliferation, differentiation, migration, survival, and repair. While Areg promotes lung branching morphogenesis, its effect on endothelial cell (EC) homeostasis in developing lungs is understudied. Therefore, we hypothesized that Areg promotes the proangiogenic ability of the ECs in developing murine lungs exposed to hyperoxia. Lung tissues were harvested from neonatal mice exposed to normoxia or hyperoxia to determine Areg expression. Next, we performed genetic loss-of-function and pharmacological gain-of-function studies in normoxia- and hyperoxia-exposed fetal murine lung ECs. Hyperoxia increased *Areg* mRNA levels and Areg+ cells in whole lungs. While *Areg* expression was increased in lung ECs exposed to hyperoxia, the expression of its signaling receptor, *epidermal growth factor receptor*, was decreased, indicating that hyperoxia reduces *Areg* signaling in lung ECs. *Areg deficiency* potentiated hyperoxia-mediated anti-angiogenic effects. In contrast, Areg treatment increased extracellular signal-regulated kinase activation and exerted proangiogenic effects. In conclusion, Areg promotes EC tubule formation in developing murine lungs exposed to hyperoxia.

## 1. Introduction

Bronchopulmonary dysplasia (BPD) is a persistent lung condition primarily seen in premature neonates. This condition is notably the leading negative outcome among premature neonates. With advancements that have enhanced the survival rate of extremely premature infants, the prevalence of BPD continues to be high [1]. The absence of specific therapies, its chronic nature, and its association with long-term cardiorespiratory and neurological complications [2,3,4,5,6] makes BPD a significantly challenging condition to manage in premature infants.

The notable histopathological features of BPD include a reduction in alveoli size and number, leading to alveolar simplification, and a reduction in and deformities of lung capillaries from disrupted lung angiogenesis, leading to pulmonary vascular simplification [7,8]. A defining characteristic of BPD is the disruption of lung angiogenesis, an essential process for normal alveolar development [9,10]. One of the reasons for the ongoing search for targeted BPD treatments is primarily due to a limited understanding of the molecular pathways involved in lung angiogenesis. Thus, there is a growing interest in molecular targets that can aid lung angiogenesis. One potential molecular avenue is the role of amphiregulin (Areg) in lung vascular development.

Areg is a growth factor of the epidermal growth factor (EGF) family that signals via the EGF receptors [11], mediating several fundamental cellular processes, including proliferation, differentiation, migration, survival, and repair. Areg is recognized primarily for its role in maintaining lung epithelial cell homeostasis [12,13,14], promoting lung branching morphogenesis [15], and facilitating wound healing, tissue repair, and regeneration [16,17,18,19]. Areg also protects against myocardial injury by promoting endothelial cell homeostasis [20]. Recently, Areg downregulation was also shown to reduce neovascularization in a mouse model of hindlimb ischemia [21]. Areg is also emerging as a key player in lung endothelial biology, influencing endothelial cell growth and survival [22,23,24,25]. However, if Areg positively influences lung endothelial health in developing lungs and, therefore, can be a target to develop BPD therapy is unclear. 

To address the knowledge gap of the effects of Areg on the developing lung vasculature, we studied the effects of hyperoxia (HO), a commonly used insult to model BPD using newborn mice [26,27,28,29] and lung endothelial cells (ECs) [30,31], on Areg expression in whole lungs and lung ECs. We also examined if and how Areg influences the effects of HO on lung angiogenesis. Specifically, we tested the hypothesis that Areg promotes the proangiogenic ability of the lung ECs in developing murine lungs exposed to HO. Our findings suggest that Areg positively influences lung angiogenesis probably by activating the enzyme, extracellular signal-regulated kinase (ERK) 2.

## 2. Materials and Methods

### 2.1. In Vivo Experiments

#### 2.1.1. Animals

This study was approved and conducted per the federal guidelines for the humane care and use of laboratory animals by the Institutional Animal Care and Use Committee of Baylor College of Medicine (Protocol# AN-5631). C57BL/6J wild-type (WT) mice (stock# 000664) were obtained from the Jackson Laboratory (Bar Harbor, ME, USA). Timed-pregnant mice raised in our animal facility were used for the experiments. 

#### 2.1.2. Hyperoxia Experiments

The HO exposure experiments were conducted with 70% blended oxygen, as mentioned before [32]. The HO group was exposed to 70% O_2_ from postnatal day (P) 1 to 14, whereas the control group remained in NO (21% O_2_) for the same time period. We avoided oxygen toxicity in the dams by rotating them daily between NO- and HO-exposed litters. 

#### 2.1.3. Lung Tissue Extraction and Real-Time RT-PCR Assays

The lungs were snap-frozen in liquid nitrogen on P14 and stored at −80 °C for subsequent RNA studies. Total lung RNA was extracted from lung tissues using the Direct-zol RNA MiniPrep Kit (Zymo Research, Irvine, CA, USA; R2052), reverse transcribed to cDNA [33], and probed using the *amphiregulin* (*Areg*; Mm01354339_m1) and *glyceraldehyde 3-phosphate dehydrogenase* (*GAPDH*; Mm99999915_g1) TaqMan gene-specific primers. *GAPDH* was used as the reference gene. The ΔΔC_t_ method was used to calculate fold changes in mRNA expression.

#### 2.1.4. Flow Cytometry Experiments

Single-cell suspensions from mouse lungs were prepared as follows. Freshly harvested mouse lungs were minced and incubated in digestion buffer (collagenase1: 5 mg/mL (Thermo Fisher Scientific; Waltham, MA, USA; catalog no. 17100-017) and DNase I: 1 mg/mL (Sigma-Aldrich; St. Louis, MO, USA; catalog no. 10104159001) in sterile PBS with Mg^+^ Ca^+^ and 0.5% BSA) for 30 min at 37 °C and agitated at 125 rpm in an orbital shaker. The cells were then dissociated by trituration through an 18-gauge needle, passed through a 100 µm strainer, and centrifuged at 300× *g* at 4 °C for 5 min. The centrifuged cells were subjected to RBC lysis for 3 min using RBC lysis buffer (BioLegend; San Diego, CA, USA; catalog no. 420301), passed through a 70 µm strainer, and centrifuged at 300× *g* at 4 °C for 5 min. The cells were later stimulated with a cell activation cocktail containing phorbol-12-myristate 13-acetate (81 nM) and ionomycin (1.34 μM) (BioLegend; San Diego, CA, USA; catalog no. 423302) for 4 h and with Brefeldin A (5 µg/mL) (BioLegend; San Diego, CA, USA; catalog no. 420601) for the last 3 h. The stimulated cells were subjected to Fc blocking (BioLegend; San Diego, CA, USA; catalog no. 156604), aqua live/dead staining (BioLegend; San Diego, CA, USA; catalog no. 423102; 1:200 dilution), and fixation and permeabilization with the eBioscience Intracellular Fixation and Permeabilization buffer set (Thermo Fisher Scientific; Waltham, MA, USA; catalog no. 88-8824-00). The stimulated, fixed, and permeabilized cells were subsequently stained with Alexa Fluor 647-conjugated anti-mouse Areg antibody (Santa Cruz Biotechnology; Dallas, TX, USA; catalog no. sc-74501; 1:50 dilution) for 40 min in perm/wash buffer to determine and quantify live Areg^+^ lung cells. Data were obtained using a Symphony flow cytometer (BD Biosciences) and analyzed with FlowJo software (Version 10.1, TreeStar, Ashland, OR, USA).

### 2.2. In Vitro Experiments

#### 2.2.1. Cell Culture

Murine fetal lung endothelial cell-like cell line, MFLM-91U, was obtained from Seven Hill Bioreagents (Cincinnati, OH, USA; catalog no. AMFLM-91U) and grown in 21% O_2_ and 5% CO_2_ at 37 °C using the EGM-2 MV Microvascular Endothelial Cell Growth Medium-2 BulletKit (Lonza Bioscience; Walkersville, MD, USA; catalog no. cc-3202), as per the manufacturer’s recommendations.

#### 2.2.2. Transient Transfection Experiments

The murine fetal lung endothelial cells were transfected with either 50 nM control small interfering RNA (siRNA) (Horizon Discovery; Cambridge, UK, catalog no. D-001810) or 50 nM target gene- and species-specific *Areg* siRNA (Horizon Discovery; catalog no. L-062462) using Lipofectamine RNAiMAX (Invitrogen; catalog no. 13778030) for at least 4 h before exposing them to hyperoxic conditions. The efficacy of siRNA transfection was validated by RT-PCR analysis and enzyme-linked immunosorbent assay (ELISA).

#### 2.2.3. Areg Treatment

The murine fetal lung endothelial cells were treated either with the vehicle, PBS, or up to 100 ng/mL of recombinant mouse amphiregulin protein (biotechne/R&D systems, Minneapolis, MN, USA, catalog no. 989-AR-100/CF) for at least 1 h before subjecting them to hyperoxia experiments.

#### 2.2.4. Hyperoxia (HO) Exposure Experiments

The murine fetal lung endothelial cells were exposed to HO (70% O_2_ and 5% CO_2_) using a ProOx110 Compact O_2_ Controller (BioSpherix, Parish, NY, USA) for up to 48 h, as we mentioned before [30].

#### 2.2.5. Real-Time RT-PCR Assays

RNA was isolated from murine fetal lung endothelial cells transfected with control and *Areg* siRNA or treated with PBS and Areg and exposed to normoxia [NO] (21% O_2_ and 5% CO_2_) or HO (70% O_2_ and 5% CO_2_) for up to 48 h. The RNA was transcribed to cDNA and probed using the *amphiregulin* (*Areg*; Mm01354339_m1), *epidermal growth factor receptor* (*Egfr;* Mm01187858_m1), and *glyceraldehyde 3-phosphate dehydrogenase* (*GAPDH*; Mm99999915_g1) TaqMan gene-specific primers.

#### 2.2.6. Enzyme-Linked Immunosorbent Assay (ELISA)

The Areg protein levels in the lung endothelial cell supernatants extracted from control siRNA- or *Areg* siRNA-transfected cells and vehicle- or recombinant Areg-treated cells exposed to NO or HO were measured and quantified by the ELISA technique using a Mouse Amphiregulin DuoSet ELISA kit (biotechne/R&D systems, Minneapolis, MN, USA, catalog no. DY989), according to the manufacturer’s recommendations.

#### 2.2.7. Immunoblot Assay

Protein lysates from recombinant Areg-treated cells exposed to NO or HO were extracted and subjected to immunoblotting, as described before [34], with anti-CD34 (Santa Cruz Biotechnology, Dallas, TX, USA; sc-7324, dilution 1:200), anti-phospho ERK 1/2 (Cell Signaling Technology, Danvers, MA, USA; 9106, dilution 1:1000), anti-total ERK1/2 (Cell Signaling Technology; 4695, dilution 1:1000), anti-GAPDH (Cell Signaling Technology; 2118, dilution 1:2000), and anti-vinculin (Cell Signaling Technology; 13901, dilution 1:8000) antibodies. The immunoreactive bands were detected and quantified [35]. Vinculin and GAPDH were used as reference proteins.

#### 2.2.8. Tubule Formation Assay

The control and *Areg* siRNA-transfected cells or vehicle-treated and Areg-treated cells were initially exposed to NO or HO. The exposed cells were then harvested and grown in 15-well µ-slide microplates (Ibidi, Gräfelfing, Germany; 81506) containing growth factor-reduced Matrigel (Corning, New York, NY, USA; 356230) at a density of 2.4 × 10^3^ cells per well for 18 h in 21% O_2_ and 5% CO_2_ at 37 °C [36,37]. The tubule formation was quantified in Matrigel after this period by Image J software (version 1.8; https://imagej.nih.gov, accessed on 22 September 2023; National Institutes of Health, Bethesda, MD, USA).

#### 2.2.9. Statistical Analyses

GraphPad Prism 10 software (GraphPad Software, La Jolla, CA, USA) was used to analyze the results. Data were tested for normality of distribution before applying the statistical tests. Data are expressed as mean ± SD. Mice exposed to NO were used as controls and compared with mice exposed to HO. Cells transfected with control siRNA or treated with PBS were used as controls and compared to Areg siRNA-transfected or Areg-treated cells, respectively, in normoxic (NO) conditions (21% O_2_ and 5% CO_2_) and hyperoxic (HO) conditions (70% O_2_ and 5% CO_2_). The effects of HO exposure on the expression of *Areg* mRNA and Areg-positive cells in the lungs were determined by the t-test if the data were normally distributed or by the Mann–Whitney test for those that failed the normality test. The effects of HO and siRNA-transfection on fetal lung endothelial cell *Areg* expression were determined by t-test, whereas the effects of *Areg* knockdown, Areg treatment, HO, and their interactions on tubule formation and Areg expression were determined by analysis of variance (ANOVA). A *p*-value of <0.05 was considered significant.

## 3. Results

### 3.1. Neonatal Murine Lung Expression of Areg mRNA and Areg^+^ Cells Following HO Exposure

**Figure 1:**
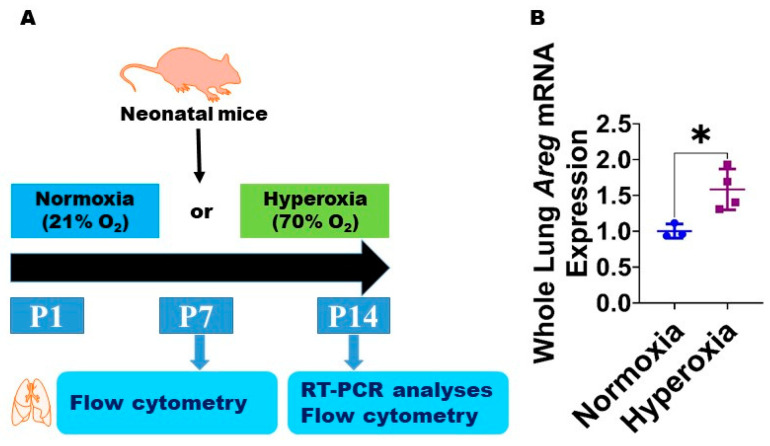
Hyperoxia (HO) exposure increases *Areg* mRNA in neonatal murine lungs. (**A**) Experimental design for Figure 1 and Figure 2. O_2_—oxygen, P—postnatal day, and RT-PCR—real-time polymerase chain reaction. Whole-lung mRNA was extracted from neonatal murine lungs after 14 d of 21% O_2_ (normoxia; n = 3) or 70% O_2_ (hyperoxia; n = 4) exposure and subjected to RT-PCR analysis to quantify *Areg* RNA expression (**B**). Data are expressed as mean ± SD. T-test was used for the statistical analyses. Significant differences between exposures are indicated by *, *p* < 0.05.

**Figure 2:**
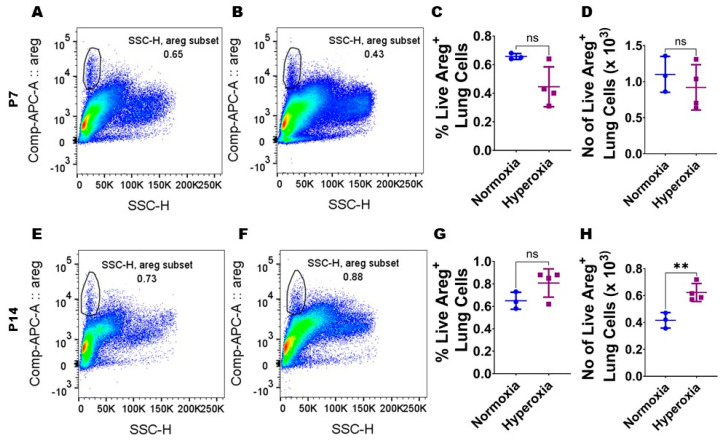
Hyperoxia (HO) exposure increases Areg^+^ cells in neonatal murine lungs. Single-cell suspensions from neonatal murine lungs exposed to 7 d or 14 d to 21% O_2_ (normoxia; n = 3/time-point) or 70% O_2_ (hyperoxia; n = 4/time-point) were extracted and subjected to flow cytometry analyses to quantify Areg^+^ cells. (**A**,**B**) Representative flow cytometry blots showing Areg^+^ lung cells from normoxia-exposed cells stained with live/dead stain and Areg antibody (**A**) and hyperoxia-exposed cells stained with live/dead stain and Areg antibody (**B**) after 7 d of exposure. (**C**,**D**) Quantification of Areg^+^ lung cell percentage (**C**) and number (**D**) after 7 d of exposure. (**E**,**F**) Representative flow cytometry plots showing Areg^+^ lung cells from normoxia-exposed cells stained with live/dead stain and Areg antibody (**E**) and hyperoxia-exposed cells stained with live/dead stain and Areg antibody (**F**) after 14 d of exposure. (**G**,**H**) Quantification of Areg^+^ lung cell percentage (**G**) and number (**H**) after 14 d of exposure. Data are expressed as mean ± SD. T-test was used for the statistical analyses. ns = not significant. Significant differences between exposures are indicated by **, *p* < 0.01.

To examine if Areg plays a role in the pathogenesis of chronic neonatal lung injury, we initially quantified the expression of *Areg* mRNA in the whole lungs from neonatal WT mice exposed to 70% O_2_ from P1 to P14, a widely established model of HO-induced experimental BPD [29]. Exposure to 14 d of HO increased the whole lung *Areg* mRNA by 1.6-fold (Figure 1B) compared with exposure to NO for a similar duration (*p*-value = 0.02). Next, we performed flow cytometry to quantify Areg^+^ lung cells at P7 and P14 using the same HO exposure experimental BPD model. At P7, the live Areg^+^ lung cell percentage (HO, 0.45 ± 0.14 vs. NO, 0.66 ± 0.02; *p* > 0.05 [Figure 2A–C]) and number (HO, 0.92 ± 0.31 × 10^3^ vs. NO, 1.1 ± 0.25 × 10^3^; *p* > 0.05 [Figure 2A,B,D]) were not statistically significantly different in neonatal mice exposed to NO and HO. Similarly, at P14, the live Areg^+^ lung cell percentage (HO, 0.81 ± 0.13 vs. NO, 0.65 ± 0.08; *p* > 0.05 [Figure 2E–G]) was similar in neonatal mice exposed to NO and HO. However, at P14, the live Areg^+^ lung cell number (HO, 0.62 ± 0.07 × 10^3^ vs. NO, 0.42 ± 0.06 × 10^3^; *p* < 0.01 [Figure 2E,F,H]) was greater in the HO group compared with the NO group. 

### 3.2. Fetal Murine Lung Endothelial Cell (EC) Expression of Areg and Its Receptor, Egfr, Following HO Exposure

**Figure 3:**
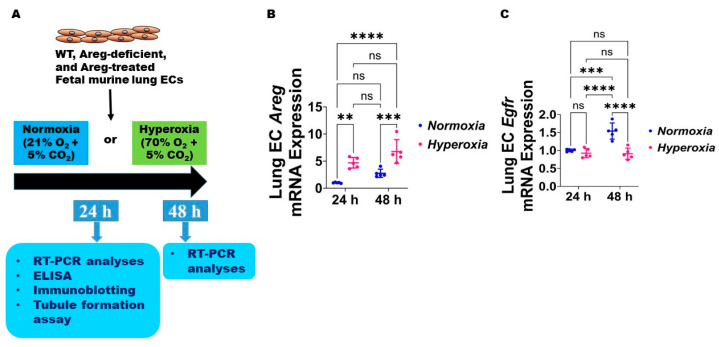
Hyperoxia (HO) exposure disrupts *Areg* signaling in fetal mouse lung endothelial-like cells. (**A**) Experimental design for Figure 3, Figure 4, Figure 5, Figure 6, Figure 7, Figure 8 and Figure 9. WT—wild type, Areg—amphiregulin, ECs—endothelial cells, O_2_—oxygen, CO_2_—carbon dioxide, RT-PCR—real-time polymerase chain reaction, and ELISA—enzyme-linked immunosorbent assay. The RNA was extracted from the fetal mouse lung endothelial-like cells exposed for 24 h or 48 h to 21% O_2_ (normoxia; n = 5/time-point) or 70% O_2_ (hyperoxia; n = 5/time-point) and subjected to RT-PCR analyses to quantify the mRNA expression of *Areg* (**B**) and *Egfr* (**C**). Data are expressed as mean ± SD. ns = not significant. Analysis of variance was used for the statistical analyses. Significant differences between exposures are indicated by **, *p* < 0.01, ***, *p* < 0.001, and ****, *p* < 0.0001.

Next, we used the commercially available fetal murine lung endothelial-like cells, MFLM-91U, to determine if Areg expression and signaling are altered in murine lung ECs following HO exposure for up to 48 h [30]. We used a similar concentration of O_2_, i.e., 70% O_2_, to maintain consistency with our *in vivo* model. The lung EC *Areg* mRNA levels increased following HO exposure for 24 h and 48 h; however, the magnitude increase in the mRNA levels following HO exposure was similar at 24 h and 48 h (Figure 3B). To determine if HO affects lung EC Areg signaling, we quantified the mRNA expression of the Areg signaling receptor, *Egfr*. Exposure to 24 h HO did not affect *Egfr* mRNA expression (Figure 3C). Continued growth of cells for 48 h increased *Egfr* mRNA expression at basal NO conditions and HO exposure for a similar duration abrogated this physiological increase in *Egfr* mRNA levels (Figure 3C). These findings suggest that prolonged HO exposure decreases Areg signaling in murine lung ECs.

### 3.3. Areg Knockdown Decreases the Tubule Formation Ability of Murine Lung ECs in HO Conditions

**Figure 4:**
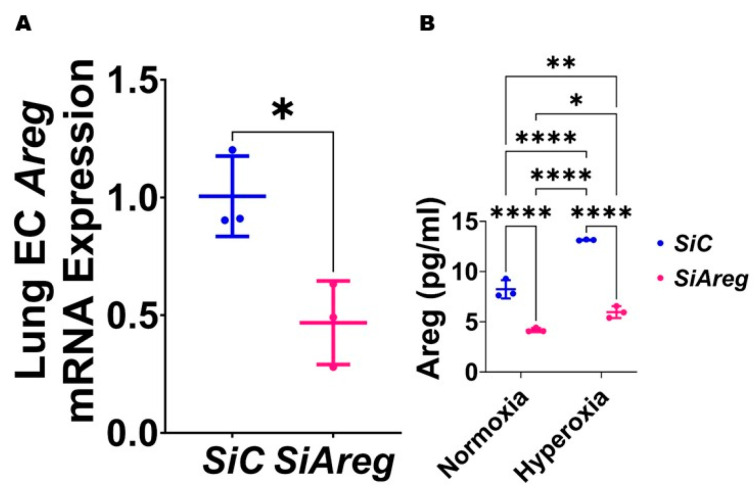
Transfection with *Areg* siRNA efficiently decreases Areg expression in fetal mouse lung endothelial-like cells. The RNA was extracted from fetal mouse lung endothelial-like cells transfected with control (n = 3) or *Areg* (n = 3) siRNA in normoxic conditions and subjected to RT-PCR analysis to quantify *Areg* mRNA expression (**A**). Values are presented as mean ± SD. T-test was used for the statistical analyses. Significant differences between exposures are indicated by *, *p* < 0.05. Subsequently, Areg protein expression was quantified by ELISA (**B**) in the cell culture supernatants of fetal mouse lung endothelial-like cells transfected with control or *Areg* siRNA and exposed to 21% O_2_ (normoxia; n = 3/group) or 70% O_2_ (hyperoxia; n = 3/group). Values are presented as mean ± SD. Analysis of variance was used for the statistical analyses. Significant differences between exposures are indicated by *, *p* < 0.05, **, *p* < 0.01, and ****, *p* < 0.0001.

**Figure 5:**
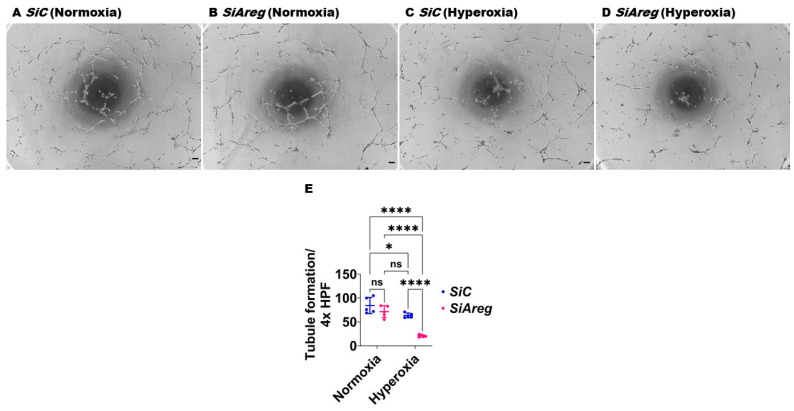
Effects of *Areg* deficiency on the tubule formation ability of fetal mouse lung endothelial-like cells. Matrigel assay was performed to quantify the tubule formation ability using fetal mouse lung endothelial-like cells transfected with control or Areg siRNA and exposed to normoxia (21% O_2_ and 5% CO_2_, n = 5/group) or hyperoxia (70% O_2_ and 5% CO_2_, n = 5/group). (**A**–**D**) Representative photographs showing tubule formation of cells transfected with control (**A**,**C**) or *Areg* (**B**,**D**) siRNA and exposed to normoxia (**A**,**B**) or hyperoxia (**C**,**D**). (**E**) Quantification of tubule formation. Scale bar = 100 µm. Data are expressed as mean ± SD. Analysis of variance was used for the statistical analyses. ns = not significant. Significant differences between exposures are indicated by *, *p* < 0.05, and ****, *p* < 0.0001.

To investigate the cause-and-effect relationship between *Areg* expression and lung EC homeostasis in HO conditions, we examined the angiogenic ability of *Areg*-sufficient and *Areg*-deficient cells upon HO exposure. To knock down *Areg*, we performed transient transfection experiments using control siRNA and *Areg*-specific siRNA and validated our knockdown by quantifying Areg mRNA and protein expression. *Areg*-specific siRNA efficiently decreased the *Areg* mRNA (Figure 4A) in NO conditions. We also confirmed that *Areg*-specific siRNA efficiently decreased Areg protein levels in cells exposed to both NO and HO conditions (Figure 4B). Having established the efficiency of *Areg* knockdown in our experimental conditions, we subsequently quantified the effects of the *Areg* gene and HO on the ability of the cells to form tubules. HO decreased the tubule formation ability of the cells (Figure 5A,C,E) and Areg deficiency potentiated the negative effects of HO on the tubule formation ability (Figure 5A,C–E). These findings suggest that *Areg* deficiency negatively affects *in vitro* lung angiogenesis upon HO exposure.

### 3.4. Recombinant Mouse Areg Protein Increases the Tubule Formation Ability of Murine Lung ECs in HO Conditions

**Figure 6:**
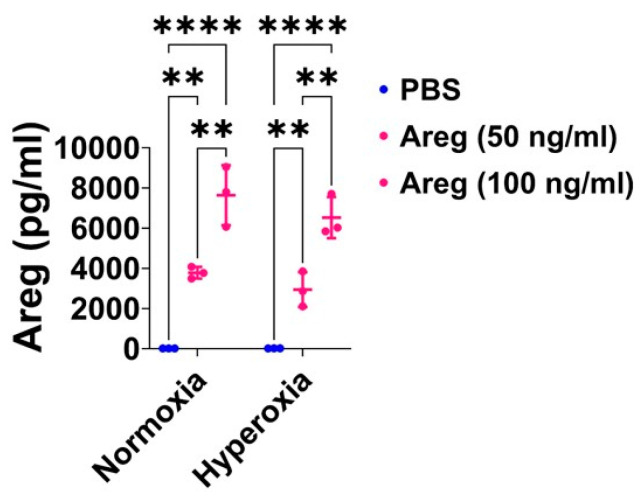
Areg treatment efficiently increases Areg protein expression in the cell culture supernatant of fetal mouse lung endothelial-like cells. Areg protein expression was quantified by ELISA in the cell culture supernatant of fetal mouse lung endothelial-like cells treated with the vehicle, phosphate-buffered saline (PBS), or up to 100 ng/mL of recombinant mouse Areg and exposed to normoxia (21% O_2_ and 5% CO_2_, n = 3/group) or hyperoxia (70% O_2_ and 5% CO_2_, n = 3/group). Values are presented as mean ± SD. Analysis of variance was used for the statistical analyses. Significant differences between exposures are indicated by **, *p* < 0.01 and ****, *p* < 0.0001.

**Figure 7:**
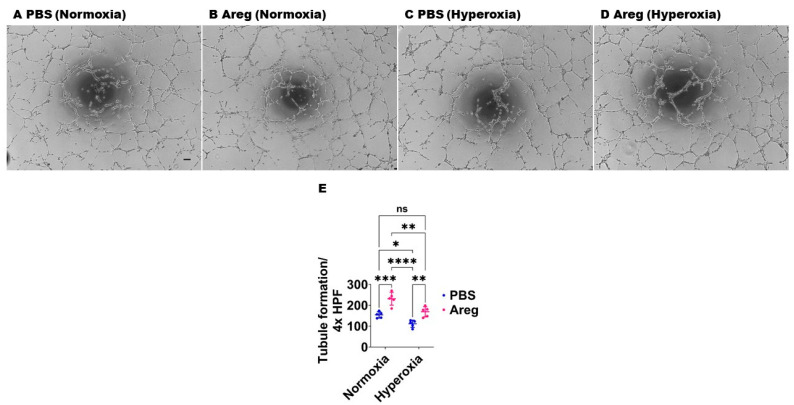
Effects of Areg treatment on the tubule formation ability of fetal mouse lung endothelial-like cells. Matrigel assay was performed to quantify the tubule formation ability using fetal mouse lung endothelial-like cells treated with phosphate-buffered saline (PBS) or 100 ng/mL of recombinant mouse Areg and exposed to normoxia (21% O_2_ and 5% CO_2_, n = 5/group) or hyperoxia (70% O_2_ and 5% CO_2_, n = 5/group). (**A**–**D**) Representative photographs showing tubule formation of cells treated with PBS (**A**,**C**) or Areg (**B**,**D**) and exposed to normoxia (**A**,**B**) or hyperoxia (**C**,**D**). (**E**) Quantification of tubule formation. Scale bar = 100 µm. Data are expressed as mean ± SD. Analysis of variance was used for the statistical analyses. ns = not significant. Significant differences between exposures are indicated by *, *p* < 0.05, **, *p* < 0.01, ***, *p* < 0.001, and ****, *p* < 0.0001.

To investigate if Areg is sufficient to improve *in vitro* lung angiogenesis upon HO exposure, we initially treated the fetal murine lung ECs with recombinant Areg and confirmed the Areg levels are increased in the treated cells both in NO and HO conditions (Figure 6). Next, we quantified the effects of Areg treatment and HO on the ability of the cells to form tubules. HO decreased the tubule formation ability of cells exposed (Figure 7A,C,E) and Areg treatment mitigated the negative effects of HO on the tubule formation ability (Figure 7A,C–E). These findings suggest that Areg treatment promotes *in vitro* lung angiogenesis upon HO exposure.

### 3.5. Recombinant Mouse Areg Protein Increases Total ERK1/2 Activation in Murine Lung ECs in HO Conditions

**Figure 8:**
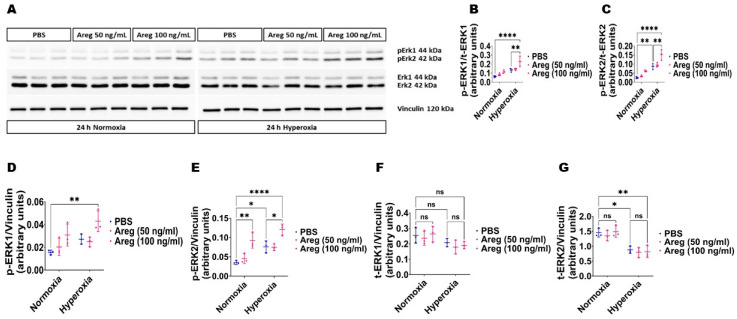
Effects of Areg treatment on ERK1/2 activation in fetal mouse lung endothelial-like cells. Whole-cell protein lysates extracted from fetal mouse lung endothelial-like cells treated with phosphate-buffered saline (PBS) or up to 100 ng/mL of recombinant mouse Areg and exposed to normoxia (21% O_2_ and 5% CO_2_, n = 3/group) or hyperoxia (70% O_2_ and 5% CO_2_, n = 3/group) were subjected to immunoblotting to quantify ERK1/2 activation. (**A**) Representative immunoblot showing the protein expression of total (t) and phosphorylated (p) ERK1/2 and vinculin. (**B**,**C**) Quantitative densitometric analyses after normalizing of p-ERK1 (**B**) and p-ERK2 (**C**) band intensities to those of t-ERK1 and t-ERK2, respectively. (**D**–**G**) Quantitative densitometric analyses after normalizing of p-ERK1 (**D**), p-ERK2 (**E**), t-ERK1 (**F**), and t-ERK2 (**G**) band intensities to those of vinculin. Data are expressed as mean ± SD. Analysis of variance was used for the statistical analyses. ns = not significant. Significant differences between exposures are indicated by *, *p* < 0.05, **, *p* < 0.01, and ****, *p* < 0.0001.

**Figure 9:**
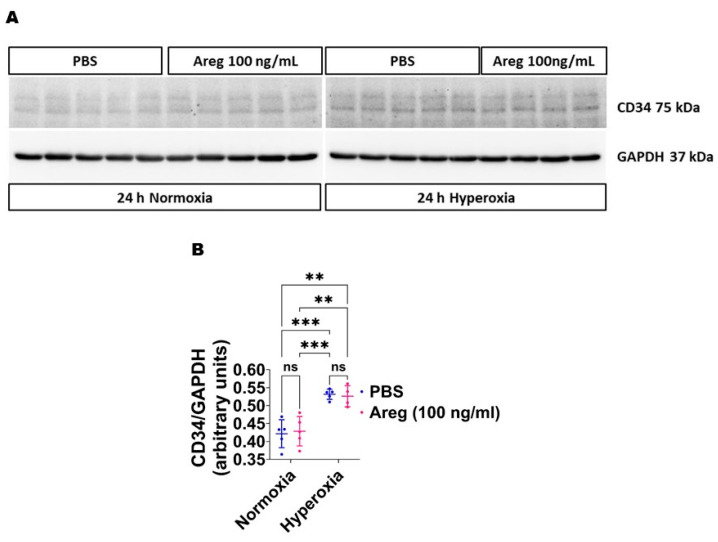
Effects of Areg treatment on CD34 protein expression in fetal mouse lung endothelial-like cells. Whole-cell protein lysates extracted from fetal mouse lung endothelial-like cells treated with phosphate-buffered saline (PBS) or 100 ng/mL of recombinant mouse Areg and exposed to normoxia (21% O_2_ and 5% CO_2_, n = 5/group) or hyperoxia (70% O_2_ and 5% CO_2_, n = 4–5/group) were subjected to immunoblotting to quantify CD34 protein expression. (**A**) Representative immunoblot showing the protein expression of CD34 and GAPDH (**A**). Quantitative densitometric analyses after normalizing of CD34 band intensities to those of GAPDH (**B**). Data are expressed as mean ± SD. Analysis of variance was used for the statistical analyses. ns = not significant. Significant differences between exposures are indicated by **, *p* < 0.01, and ***, *p* < 0.001.

Because many growth factors mediate their downstream cellular effects via ERK1/2 and ERK2 to promote EC health in developing murine lungs [34,38], we investigated if Areg promotes murine lung EC tubule formation via ERK1/2 activation. Exposure to HO alone increased ERK2 activation, as evidenced by increased p-ERK2 expression (Figure 8A,C,E) and decreased t-ERK2 expression (Figure 8A,G) in HO conditions. However, the extent of HO-induced ERK2 activation was significantly greater in Areg-treated cells than in vehicle-treated cells (Figure 8A,C,E). Further, Areg treatment increased p-ERK1 activation in HO conditions (Figure 8A,B,D). These findings suggest Areg increases total ERK1/2 activation in HO-exposed murine lung ECs. We also investigated if Areg affects CD34 protein expression in this *in vitro* model. Although HO increased CD34 protein expression, Areg treatment did not independently affect the expression of this protein (Figure 9).

## 4. Discussion

In our research, we investigated how hyperoxia influences the levels of *Areg* mRNA and the presence of Areg^+^ cells in neonatal murine lungs and the interaction between hyperoxia and Areg on fetal murine lung endothelial cell homeostasis using clinically relevant *in vivo* and *in vitro* models. Our findings indicate that hyperoxia exposure increases *Areg* mRNA levels and Areg^+^ cell numbers in neonatal murine lungs *in vivo*. Further, we demonstrate that while hyperoxia augments the mRNA levels of *Areg*, it reduces the levels of its signaling receptor, *Egfr*, in fetal lung endothelial cells, suggesting that hyperoxia decreases *Areg* signaling in the lung endothelial cells. Finally, through a series of loss-of-function and gain-of-function studies, we further establish that Areg positively influences lung endothelial cell angiogenesis in association with ERK1/2 activation.

Areg promotes angiogenesis [22,23,24,25] and mitigates PH [22] and myocardial ischemic reperfusion injury [20] in adult rodents. These observations indicate Areg is important for cardiopulmonary health. However, the role of Areg in experimental BPD is understudied. Hence, we initially quantified the lung *Areg* mRNA levels and Areg+ cells in our mouse model of experimental BPD that closely models the short-term and long-term cardiorespiratory morbidities, including disrupted angiogenesis, seen in BPD infants [28,29]. In agreement with prior studies [39,40,41], we show that HO increases *Areg* mRNA expression in neonatal rodent lungs. Further, we demonstrate that HO increases Areg^+^ lung cells. Yao et al. [42] recently also showed increased type 2 innate lymphoid^+^ Areg^+^ cells in HO-exposed neonatal murine lungs. In the lungs, Areg is also expressed in the endothelial, epithelial, smooth muscle, and mesenchymal cells [15,22,43,44,45,46,47] and in resident regulatory T cells [14,48]. Whether HO also increases Areg expression in the ECs of developing lungs is not well characterized. We focused on Areg signaling in ECs since lung ECs maintain lung homeostasis and alveolar health across the lifespan. In human and experimental BPD characterized by alveolar simplification, the expression of angiogenic molecules [49,50,51,52,53] and the extent of angiogenesis [7,8,9,49,54,55,56,57,58,59] are decreased.

To determine the role of Areg signaling in the developing lung ECs, we used the fetal murine lung endothelial cell line, MFLM-91U, because it has been widely used to study the effects of HO on the EC biology of developing murine lungs in a robust manner [60,61,62]. Consistent with our *in vivo* findings in whole lungs, HO increased *Areg* mRNA expression in the lung ECs. As a growth factor, Areg mediates its biological effects in an autocrine and paracrine manner. Epidermal growth factor (EGF) receptors are expressed in epithelial cells of the airway and alveoli [43,44], while Areg is expressed in ECs and epithelial, smooth muscle, and mesenchymal cells [15,22,43,44,45,46,47] and resident Tregs [14,48]. To determine if Areg can mediate its effects in an autocrine manner in our *in vitro* model, we determined the expression of its signaling receptor, *Egfr*, in the fetal murine lung ECs. We demonstrate that fetal murine lung ECs express the Areg receptor. Further, we show that HO inhibits *Areg* signaling in these ECs, as evidenced by reduced *Egfr* mRNA levels under hyperoxic conditions. Previous studies have also reported reduced Areg and Egfr expression in the lung ECs of adults diagnosed with PH [22]. Hence, our findings reinforce the concept that Areg signaling could play an important role in lung EC biology even in developing lungs. 

To discern whether the decline in Areg signaling is either a causative or an adaptive event in the HO-driven disruption of EC homeostasis, we performed loss-of-function and gain-of-function studies. We noted that *Areg* deficiency inhibited while recombinant Areg treatment promoted fetal murine lung EC angiogenesis under hyperoxic conditions. These findings support our hypothesis that Areg promotes the proangiogenic ability of the lung ECs in HO-exposed developing lungs. Several studies have highlighted the angiogenic effects of Areg in mature lungs, particularly in the context of cancer and allergic and inflammatory disorders [22,23,24,25]. Our results align with these studies, revealing that a similar phenomenon occurs in HO-exposed developing lungs. Importantly, we show that Areg acts in an autocrine manner in ECs, highlighting the importance of EC homeostasis in maintaining lung health. Our findings also complement the beneficial role of Areg in the epithelial biology of developing lungs [15]. Therefore, our findings have important implications in the prevention and treatment of BPD, a disorder characterized by hindered lung angiogenesis and alveolarization.

Finally, to understand the pathways through which Areg exerts its angiogenic influence, we examined the impact of recombinant Areg treatment on ERK1/2 activation because growth factors acting via Egfr predominantly mediate their effects via ERK1/2. Existing research has highlighted that Areg augments ERK1/2 activation in mature lung epithelial cells [63,64,65], aortic smooth muscle cells [66], bone tissues [67], keratinocytes [68], and breast [69] and pancreatic [70] cancer cells. Yet, our data reveal that Areg similarly stimulates ERK1/2 in developing lung ECs. Further, we previously demonstrated that ERK1/2 activation positively influences lung EC homeostasis in developing lungs [31,34,38]. CD34^+^ cells maintain tissue homeostasis [71] and are activated during tissue injury, increasing the expression of growth repair tissue factors such as Areg [72]. CD34 is also an angiogenic marker, and HO exposure has dual effects on the expression of this protein [73,74]. Our findings indicate that HO but not Areg increases CD34 expression in fetal murine lung ECs. Since CD34 influences Areg expression [72], it needs to be investigated if HO increases Areg expression via CD34. Our findings suggest that Areg promotes lung EC homeostasis predominantly via ERK1/2 activation. Areg may influence EC health via several other mechanisms, including BCL2-associated agonist of cell death [22], inflammatory cell polarization [21], and vascular endothelial growth factor A [75]. 

A significant constraint of our research is that the direct relationship between *Areg* signaling and lung angiogenesis was primarily assessed through *in vitro* experiments. Nonetheless, our *in vivo* investigations using a clinically pertinent model of experimental BPD suggest a comparable interplay. We aim to rectify this shortcoming in our future research by conducting genetic and pharmacological loss-of-function and gain-of-function *in vivo* experiments to validate the therapeutic potential of Areg in experimental BPD.

## 5. Conclusions

**Figure 10:**
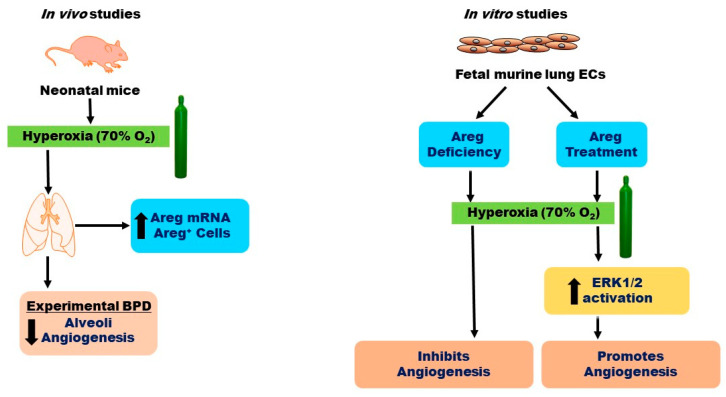
Overview of the results. O_2_—oxygen, BPD—bronchopulmonary dysplasia, ECs—endothelial cells, Areg—amphiregulin, and ERK—extracellular signal-regulated kinase.

In conclusion, our findings reveal that HO exposure elevates both *Areg* mRNA levels and the number of Areg^+^ cells in the lungs of neonatal mice *in vivo* (Figure 10). Additionally, we have determined that while HO amplifies *Areg* mRNA levels, it reduces *Egfr* mRNA levels, leading to diminished *Areg* signaling in fetal murine lung ECs. Ultimately, we have ascertained that Areg fosters lung EC tubule formation and ERK1/2 activation in hyperoxic conditions (Figure 10). To the best of our knowledge, this is the first *in vitro* study to investigate the effects of Areg on the ECs in developing lungs. Our findings provide a rationale for conducting mechanistic studies targeting Areg in experimental BPD models. Using cell-specific, *Areg*-deficient, and -overexpressing mice to determine the direct effects of Areg on lung alveolarization, vascularization, and pulmonary hypertension in experimental BPD models could inform us of Areg’s therapeutic potential for infants with BPD.

## Data Availability

The data presented in this study are available in the article.

## Abbreviations

Areg—amphiregulin, BPD—bronchopulmonary dysplasia, ECs—endothelial cells, EGF—epidermal growth factor, Egfr—epidermal growth factor receptor, ERK—extracellular signal-regulated kinase, ELISA—enzyme-linked immunosorbent assay, GAPDH—glyceraldehyde 3-phosphate dehydrogenase, HO—hyperoxia, MFLM—murine fetal lung endothelial cell-like cell line, NO—normoxia, PBS—phosphate-buffered saline, SiC—control siRNA, SiAreg—Areg siRNA, WT—wild-type.
